# Green algae scatter off sharp viscosity gradients

**DOI:** 10.1038/s41598-020-79887-7

**Published:** 2021-01-11

**Authors:** Simone Coppola, Vasily Kantsler

**Affiliations:** Physics Department, University of Warwick, Coventry, CV47AL UK

**Keywords:** Biological physics, Motility

## Abstract

We study the behaviour of the green alga *Chlamydomonas reinhardtii* (CR) in the presence of neighbouring regions of different viscosity. We show that the velocity and angular diffusion of the algae decreases when the viscosity of the surrounding medium is increased. We report on a phenomenon occurring when the algae try to cross from a region of low viscosity to a highly viscous one, which causes CR to re-orient and scatter away from the interface if it is approached at a sufficiently small angle. We highlight that the effect does not occur for CR crossing from high to low viscosity regions. Lastly we show that algae do not concentrate in the region of high viscosity despite them swimming slower there. On the contrary, they concentrate in the region of low viscosity or maintain a uniform concentration profile, depending on the viscosity ratio between the two regions.

## Introduction

In nature, micro-organisms often have to respond to external stimuli and changes in their surroundings when navigating through an environment. These stimuli can be of various sorts, from chemical gradients^1^ to external flows^2^ or even topological gradients^3^.

Another change in the environment that is being navigated by the micro-organisms can be the viscosity of the medium in which they are swimming. It is indeed quite common to find a variety of natural environments which have spatially inhomogeneous viscosities, ranging from sedimentation profiles and biofilms to the female reproductive tract^4,5^.

Recently, theoretical frameworks have been developed to predict the behaviour of a microswimmer in a viscosity gradient. For example Liebchen et al.^6^ showed how certain body shapes can lead to negative viscotaxis (i.e. moving away from a high viscosity region) due to a systematic imbalance of the viscous forces acting on the microswimmer. Similarly, Datt and Elfring^7^ demonstrated that the response of a microswimmer to a viscosity gradient depends on its swimming gait and that different gaits can lead to negative or positive viscotaxis, based on hydrodynamics.

**Figure 1:**
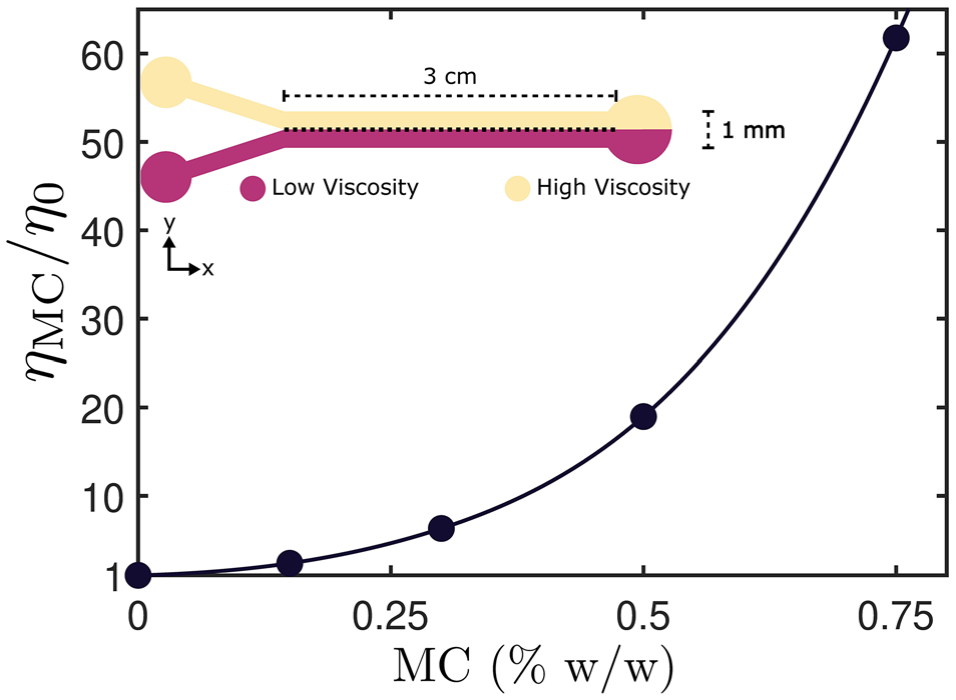
Dynamic viscosity measurements for the various solutions of DI Water and methylcellusose which were used as high viscosity media. The circles indicate the concentrations used during the experiments: 0.15%, 0.30%, 0.50% and 0.75%. In the inset, the geometry of the microfluidic device used to perform the experiment (inlets on the left, outlet on the right). Through laminar flow and balanced flow rates it is possible to generate two regions of equal width but different media, as highlighted by the different colours. The device schematic was drawn and added to the figure using the proprietary software Affinity Designer 1.8 (https://affinity.serif.com/en-gb/designer/).

In order to carry out an experimental investigation on the matter we perform experiments using microfluidics devices, which have proven over recent years to be the standard tool when performing systematic research on microbial motility in inhomogeneous environments due to their low price and ease of use^1,8^. We perform our research using a device which generates a sharp viscosity gradient (i.e. two adjacent regions of significantly different viscosity) which we believe can help identify whether there is a major effect to be seen and investigated further (Fig. 1). We also note that we will be using the terms “sharp gradient” and “interface” interchangeably in the manuscript.

We use *Chlamydomonas reinhardtii* (CR), a biflagellate $10\,\upmu \text {m}$ “puller” microswimmer which achieves locomotion through breaststroke-like beating and has been thoroughly studied and accepted as a model biological microswimmer in recent years^9^.

Qin et al. ^10^ first showed experimentally that the motility of CR is strongly influenced by the viscosity of the environment which they inhibit. In fact, the motility of microorganisms swimming in viscous environments is determined by a complex interplay between the swimmers’ kinematics and material properties as well as the properties of their surrounding environment. Such interplay can include aspects such as the increased drag on the flagella, their rigidity as well as any mechanosensory response from the microswimmers i.e. passive and active responses to the environment^5^.

Viscous environments can therefore have profound effects on the propulsion speed and beating frequency of the algae. However, this study by Qin et al.^10^ is only concerned with environments of homogeneous viscosity, and only explores the effects of viscous environments up to $\sim 10$ times more viscous than water.

Viscosity has also been predicted to play a role in the flagellar dynamics of CR by Klindt et al.^11^, who have showed that flagellar synchronization is dependent on the viscosity of the medium in which CR is swimming. In fact, while free swimming CR usually display in-phase synchronization of their flagella to achieve their breaststroke-like swimming, it is also possible for the flagella to lock in an anti-phase synchronization^12^. In such cases, the flagella motion resembles that of freestyle swimming and is characterized by straighter trajectories, as opposed to the run-and-tumble motion which results from breaststroke-like swimming^13^.

In this manuscript we observe how wild-type (wt) and short-flagella (sfl) CR respond to viscous environments by diluting Methylcellulose (MC) in water (see “Methods ” section). This allows us to produce media with dynamic viscosity $\eta _{\text {MC}}$ up to $\sim 60$ times that of water $\eta _{\text {0}} = 1.0016 \text { cP}$ (Fig. 1).

We choose to investigate the two strains as there have been experimental reports which suggest the effects of increasing viscosity could change depending on the flagellar length of the algae^14^. We analyse how the velocity and angular diffusion of the algae change as the viscosity of the medium is increased, expanding previously published experimental results and predictions made on the matter^10^.

We also perform an analysis of single cell trajectories for CR approaching the interface between the low and high viscosity region, showing that algae can scatter off the interface if they approach it from the low viscosity region at a sufficiently small angle—therefore confirming previous suggestions that “puller” microswimmers could exhibit viscophobicity due to torque imbalance^6,7^.

Finally, we explore how the combination of these different phenomena affects the concentration profile of CR in the channel. Surprisingly, we find that CR either concentrate in the low viscosity region (i.e. where they swim faster) or nowhere at all, maintaining the concentration profile in the chamber uniform. This highlights that the velocity distribution in the chamber is not the main factor dominating how CR diffuse in the chamber, but rather that the results are a combination of all the effects previously mentioned.

## Results and discussion

### Velocity distributions and motility characterization

**Figure 2:**
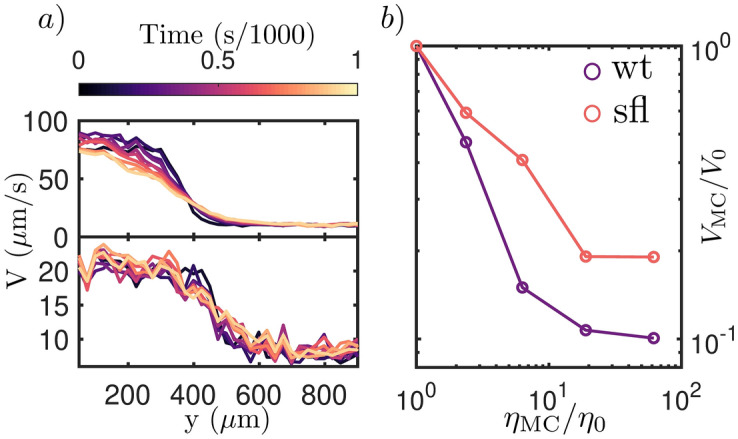
(**a**) Swimming velocity of wild-type (top) and short flagella (bottom) *Chlamydomonas reinhardtii* algae as a function of their position in the device in experiments in which 0.30% MC ($6\eta _{0}$) is used for the high viscosity region between 0 and 1000s after the syringe pump is stopped at the beginning of the experiment (see Fig. 1 for a coordinate system reference), showing that the gradient is stable for the time of interest. (**b**) The velocity ratio $V_{\text {MC}}$/$V_{\text {0}}$ as a function of the concentration of MC in the high viscosity region. Figure panels were arranged and labels were added using the proprietary software Affinity Designer 1.8 (https://affinity.serif.com/en-gb/designer/).

Figure 2a shows how the velocity of CR changes as a function of its position in the device when 0.30% MC ($6\eta _{0}$) is used to generate the high viscosity region. As expected, the device generates two regions where the velocity of the algae is significantly different. Furthermore, we measure how the swimming velocity distribution *V*(*y*) across the microfluidics device changes as a function of time. This provides a way to indirectly keep track of how stable the gradient is during the experiment, since a smoothing of the gradient is to be expected due to methylcellulose diffusing. We show the gradient is stable for about 1000s.

In order to fully understand how different viscosities affect the swimming velocity of wild-type (wt) and short-flagella (sfl) CR, the ratio $V_{\text {0}}$/$V_{\text {MC}}$, where $V_{\text {0}}$ is the velocity of CR in its regular medium and $V_{\text {MC}}$ the velocity in the high viscosity medium, is measured for different concentrations of MC in DI water (Fig. 2b).

The results shown in Fig. 2 agree with previous findings reported by Qin et al.^10^ for wt Chlamydomonas, which show that the velocity of swimming algae decreases approximately as $1/\eta$ for viscosity values up to $\sim 10\eta _{\text {0}}$. This result, as already highlighted by Qin et al.^10^, indicates the algae continue swimming with the same thrust despite the increased viscosity of the media.

We expand on these previous findings by measuring the velocity of CR in environments up to $60\eta _{0}$, showing both strains of CR do not decrease their velocity any further for viscosities of $\sim 30\eta _{0}$ and higher. Such results suggest that for sufficiently high viscosities ($>10\eta _{0}$) CR might change its swimming gait to optimize efficiency, similar to what has been reported for sperm cells swimming in high viscosity environments^5^.

Interestingly, we also observe that increasing the viscosity has a larger effect on wt CR than the sfl strain. To understand this peculiar finding, we refer to the report published by Bottier et al.^14^, who carried out an in depth investigation of how cilia beating is affected by its length. In particular, the authors investigated how the force required to overcome viscous drag changes as a function of flagellar length. Results showed that the force required to overcome drag increases with the length of the flagella, while the overall dynein force (i.e. the force moving the flagella) does not. While this experimental investigation only concerns algae swimming in regular viscosity media and a more detailed study would be needed to confirm our suspicion, we believe its results can offer a qualitative explanation for the difference in effect we measure between the two CR strains.

To characterize the behaviour and motility of CR in high viscosity media, we determine the translational ($D_{\text {T}}$) and rotational ($D_{\text {R}}$) diffusivity of the algae by calculating the mean-squared displacement (MSD) and mean-squared angular displacement (MSAD) from their trajectories.

The MSD is defined as $\text {MSD}(\Delta t)=\langle | \mathbf {r}(t_{0}+\Delta t) - \mathbf {r}(t_{0}) |^{2} \rangle$. CR exhibit ballistic behaviour at short timescales ($\text {MSD} \propto \Delta t^{2}$), while at sufficiently long scales their motion can be characterized as diffusive ($\text {MSD} \propto \Delta t$). Therefore, for CR performing a random walk in two dimensions, it is possible to extract $D_{\text {T}}$ from $\text {MSD}(\Delta t) = 4D_{\text {T}}\Delta t$.

**Figure 3:**
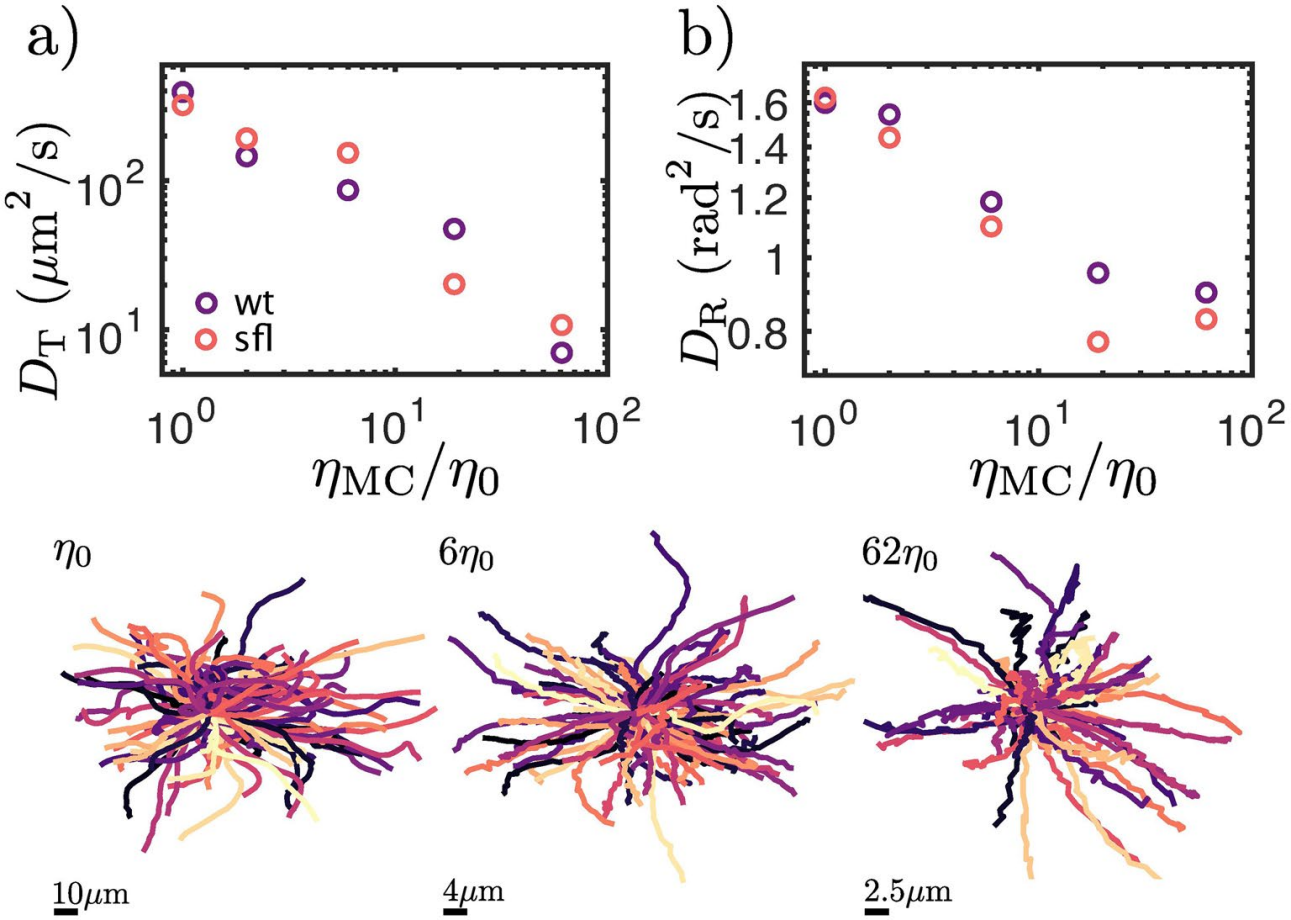
Top row: translational and rotational diffusion coefficients for wt and sfl CR as a function of the viscosity of the surrounding medium. Bottom row: typical 3s trajectories for CR swimming in different viscosity media. Figure panels were arranged and labels were added using the proprietary software Affinity Designer 1.8 (https://affinity.serif.com/en-gb/designer/).

Figure 3a shows the $D_{\text {T}}$ extracted values for wt and sfl CR swimming in environments of increasing viscosity. As expected from the decrease in velocity observed in Fig. 2, the translation diffusivity of CR also decreases as the viscosity of the surrounding environment is increased.

The MSAD is defined $\text {MASD}(\Delta t) =\langle \left| \phi (t_{0}+\Delta t) - \phi (t_{0}) \right| ^{2} \rangle$. In this case, all curves showed a diffusive behaviour, making it possible to extract the rotational diffusivity value from $\text {MASD} = 2D_{\text {T}}\Delta t$. Figure 3b shows the rotational diffusivity of CR decreases when the viscosity of the surrounding medium is increased. This is further highlighted by the typical trajectories (Fig. 3), in which it possible to observe that CR swimming at high viscosity do not display any sharp changes in direction, but are rather straight. This contrasts with typical CR trajectories for algae swimming in low viscosity media, which have been observed to display a “run-and-tumble” type of motion.

It is possible to explain the decrease in angular diffusivity and the change in trajectory shape by considering the effect the surrounding viscosity has been predicted to have on CR flagellar beating and synchronization. Klindt et al.^11^ have predicted that a high viscosity medium could induce anti-phase synchronization of the flagella i.e. freestyle swimming as opposed to the traditional breaststroke swimming which is observed at low viscosity.

### Scattering at the interface

After measuring how the velocity and diffusion properties of CR change in the two regions, we study the behaviour of the algae as they approach the interface. In order to avoid any bias from the diffusion of the gradient, we only consider trajectories obtained in the first 100s after setting up the two regions. We then consider how the scattering angle $\theta _{\text {out}}$ varies as a function of the incoming angle $\theta _{\text {in}}$ for CR trying to cross the interface from the low viscosity region to the high viscosity region and viceversa (see Supplementary Materials Fig. S1 for more details). We define $\theta _{\text {in}}$ and $\theta _{\text {out}}$ as the average orientation of CR 1s prior to reaching and leaving the interface respectively.

**Figure 4:**
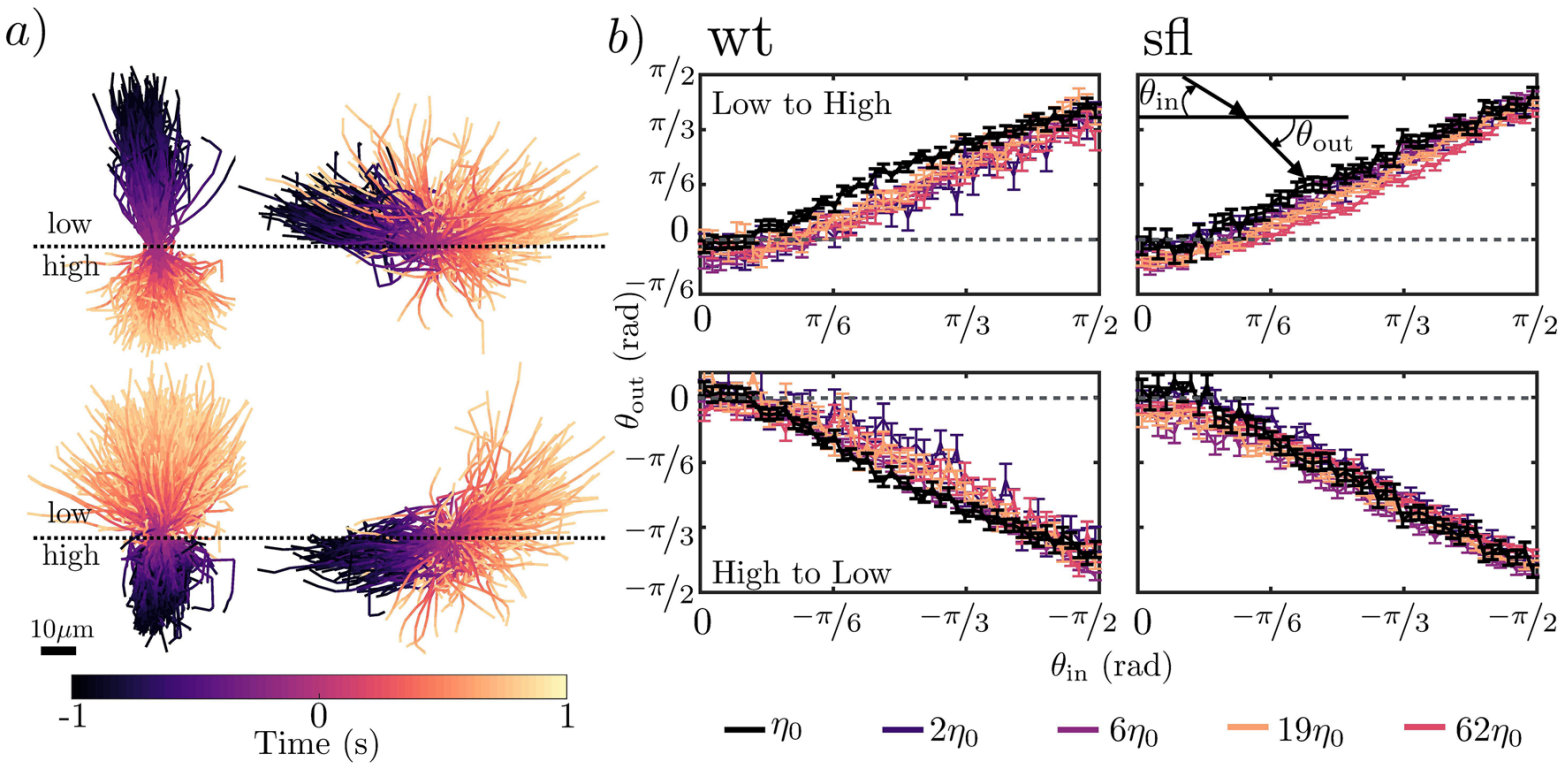
Left: examples of low and high incoming angle trajectories for wt CR trying to cross from low to high (0.30% MC, $6\eta _{\text {0}}$) viscosity region and viceversa; Right: resulting outgoing angle for a given incoming angle when CR try to cross the interface. The black line indicates a control experiment, in which algae are crossing from the low viscosity region to an identical one. For each line, $\sim 10^{5}$ crossings were recorded and analysed. Figure panels were arranged and labels/legends were added using the proprietary software Affinity Designer 1.8 (https://affinity.serif.com/en-gb/designer/).

When algae try to cross the interface to go from the low viscosity region to high viscosity one, we find that for small incoming angles ($\theta _{in}\lesssim \pi /6$) both wt and sfl CR tend to scatter off it and stay in the low viscosity region. On the contrary, when approaching the interface from the high viscosity region, CR manage to cross to the low viscosity region. Furthermore, for the cases in which $\eta _{\text {MC}}/\eta _{\text {0}}>1$, we find $\theta _{\text {out}} (\theta _{\text {in}})$ to have no significant dependence on the viscosity ratio between the two regions or the length of the flagella (Fig. 4b). Figure 4a shows trajectories for wt CR approaching the interface for small and large incoming angles. This allows to visualise quite clearly how CR scatters off the interface for small angles when trying to cross from low to high viscosity but can cross successfully in the opposite case. Interestingly, the scattering effect we observe resembles that of crawling cells scattering away from high friction regions when trying to cross from a low friction one^15,16^.

We can understand the origin of these experimental results by using the theoretical model proposed by Datt and Elfring^7^ in their work. The authors have in fact shown that a squirmer swimming through a region of linearly changing viscosity gradient will have an angular velocity of the form $\varvec{\Omega } = \frac{1}{2}\mathbf {U}_{N} \times \mathbf {\nabla }(\eta / \eta _{\text {0}})$, where $\mathbf {U}_{N}=\left( \frac{2B_{1}}{3}\right) \mathbf {e}$ is the velocity of a squirmer in a Newtonian fluid of uniform viscosity and $B_{1}$ and $\mathbf {e}$ represent the first squirming mode coefficient and the orientation of the microswimmer (full derivation and details can be found in the referenced work). We can then consider—similar to what has been done in^7^—the case in which the gradient increases linearly only in one direction. It is then immediately obvious that the torque caused by the change in viscosity will be at its greatest for the case $\mathbf {e} \perp \mathbf {\nabla }(\eta / \eta _{\text {0}})$ and will tend to zero as $\mathbf {e} \parallel \mathbf {\nabla }(\eta / \eta _{\text {0}})$.

It is however necessary to point out that Datt and Elfring’s model is based on two assumptions: (i) the squirmers are freely swimming and are not bound by any surfaces and (ii) the gradient is linear and slowly changing. Our experiments—as outlined in the “Methods” section—are performed in microfluidic chambers of $\sim 20\upmu \text {m}$ height and thus the algae examined in this paper are not freely swimming but rather confined. This confinement has been shown to affect the properties of the flow field generated by CR algae both theoretically and experimentally^17–20^, therefore a direct comparison between our work and that of Datt and Elfring is not possible. Similarly, as we mentioned in the previous sections and in the “Methods” section, the type of gradient we study in this paper is a sharp gradient rather than a linearly increasing one. Nevertheless, we do believe the model outlined can offer a way to qualitatively understanding our results, particularly in the context of the concentration profiles analysed in the next section of this manuscript.

The equations of motion derived by Datt and Elfring et al.^7^ for a squirmer in a viscosity gradient can also be used to gain some intuitive understanding as to why there is critical angle above which algae can cross successfully over a sharp interface. In fact, as the incoming angle $\theta _{\text {in}}$ approaches $\pi /2$ the swimmer will navigate deeper into the gradient before re-orienting towards the lower viscosity region. Since in our case the region of changing viscosity is of a finite length, results suggest that for angles over $\sim \pi /6$, algae can successfully reach the high viscosity region.

Lastly it is worth pointing out that while Datt and Elfring include the type of swimming gait in their work distinguishing between pushers and pullers, they do not include any potential change to swimming gait due to viscosity or viscous forces on individual flagella, which are likely to play a significant role in the effects reported in this manuscript.

### Concentration profile

Lastly, we studied the time evolution of the concentration profile of the algae in the chamber. Measurements were taken for 1000s, as it was established the gradient is stable in this timescale (Fig. 2). In order to quantify where the algae concentrate in the device we introduce the normalised ratio $\nu$, which is defined as $\nu =\text {(}N_{\text {H}}-N_{\text {L}}\text {)/(}N_{\text {H}}+N_{\text {L}}\text {)}$, where $N_{\text {H}}$ and $N_{\text {L}}$ are the number of algae in the high and low viscosity regions respectively (more information in Figs. S2 and S3 of the Supplementary Material).

Interestingly, we observe that wt CR concentrate in the low viscosity region for experiments with high viscosity of $2\eta _{0}$ and $6\eta _{0}$, whereas the concentration remains uniform for the $19\eta _{0}$ and $62\eta _{0}$ cases. Similarly, sfl CR tend to concentrate in the low viscosity region. The results are particularly striking because recent studies have shown that it is possible to concentrate microswimmers in regions where their velocity is lower, due to the increased residence time. However, it is worth noting that the method of generating a velocity distribution in this paper is significantly different than those employed in those studies, in which photokinetic bacteria were used^21,22^.

In summary, we believe the results to be a combination of all the effects mentioned in the previous sections: (i) the velocity distribution, which favours concentration in regions where the algae swim slower; (ii) the decrease in angular diffusion for algae in high viscosity media; (iii) the re-orientation effect at the interface, which can help algae accumulate at low viscosity, since it will create a flux imbalance of algae in favour of the low viscosity region.

**Figure 5:**
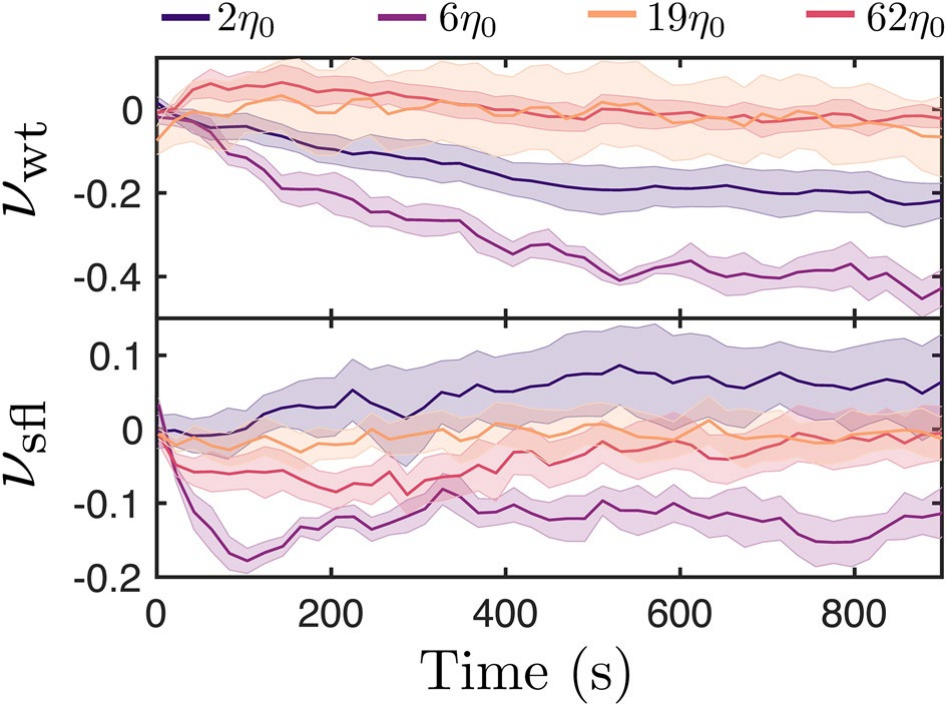
The time evolution of the normalised ratio $\nu$ for wt (top) and sfl (bottom) CR for different viscosity ratios of the two regions. In all cases there is no accumulation in the high viscosity region ($\nu \rightarrow 1$), but rather accumulation in the low viscosity region or nowhere at all. Labels and legend were added using the proprietary software Affinity Designer 1.8 (https://affinity.serif.com/en-gb/designer/).

It is possible for the effects to cancel each other out, keeping the concentration profile uniform in the channel. Similarly, the effects can lead to accumulation in the low viscosity region, as is it shown in Fig. 5.

Lastly, as mentioned in the introductory section, microswimmer response to viscous environments is caused by a complex interplay of active (mechanosensory) responses as well as passive ones (i.e. the increased drag on the flagellum and the body or the flow field signature of the microswimmer). Consequently, the results reported are valid for CR algae but are likely to differ for other types of microswimmers, such as sperm cells (pushers instead of pullers), which have been reported to have a similar swimming velocity in low and high viscosity fluids^5^ and would cross the interface differently due to their flow field, as predicted by Datt and Elfring^7^.

## Methods

### Algae culture

Liquid cultures were grown axenically in a $20 ^\circ \text { C}$ diurnal chamber set on a 16/8 hour day/night cycle on a rotary shaker set at $\sim 100\text { rpm}$.

The cultures were kept at a population density of $10^{5}$ cells/ml, so that experiments could be performed with algae in their exponential growth stage.

### Microfluidics and microscopy

Polydimethylsiloxane (PDMS) Y-junction microfluidics devices ($\sim 20\,\upmu \text {m}$ high) were manufactured via standard soft lithography techniques and bonded to glass coverslips after treatment in a plasma chamber (Harrick). To achieve optimal bonding the devices were left for 2 minutes on a hot plate set at $80^\circ \text { C}$ immediately after plasma cleaning.

The device was used to generate two bands of equal width but significantly different viscosity through the use of two syringe pumps equipped with $100\,\upmu \text {l}$ gastight syringes (Hamilton). Two syringe pumps (KD Scientific) were used to balance the flow rates depending on the viscosity ratio of the two fluids employed in order to generate bands of equal size.

High viscosity media were prepared by dissolving different concentrations (0.15%, 0.30%, 0.50% and 0.75%) of high-grade Methylcellulose (MC) (Sigma Aldrich, M0512) in DI water and an Ubbelohde viscometer was used to measure the dynamic viscosity of the different solutions (Fig. 1). The viscosity measurements were found to be in agreement with previously published results^23^, which also show that for the concentration range used in this paper MC behaves as a Newtonian fluid.

In order to obtain equal concentrations of CR in both media used in the experiments, two 1.5 ml eppendorfs were filled with cultures and centrifuged at $\sim 1.2 \text {k}$ rpm for 12 min. The supernatant was then removed and substituted with DI water (low viscosity medium) and one of the MC solutions (high viscosity medium). The two eppendorfs were then placed on a rotary shaker for 30 min to ensure that any deflagellated algae would regrow their flagella before filling the microfluidic device and performing the experiment. The cell concentration at which experiments were performed prevents any collective hydrodynamics effects to affect the results reported. All experiments were performed at room temperature.

Brightfield microscopy was performed using a Nikon Eclipse TE2000U inverted microscope equipped with a 4$\times$ objective (Nikon, NA 0.10). To prevent any phototactic bias, a longpass coloured glass filter (RG715, Thor Labs) was used to block any wavelength below $715\text { nm}$. Image frames (10 fps) were recorded using a 1920 $\times$ 1200 pixel CCD camera (Pointgrey Grasshopper3) and processed through custom MATLAB software developed to extract microswimmer trajectories and analyse them.

## Supplementary information

Supplementary information.

## Data Availability

The datasets generated during and/or analysed during the current study are available from the corresponding author on reasonable request.
