# Supplementary material for: Green algae scatter off sharp viscosity gradients
Source: Sci Rep. 2021 Jan 11;11:399. doi: 10.1038/s41598-020-79887-7 (PMC7801662; doi:10.1038/s41598-020-79887-7)
Supplement: Supplementary file 1 — Supplementary information. [file 41598_2020_79887_MOESM1_ESM.pdf]

Supplementary material for *Green algae scatter off sharp viscosity gradients*

S. Coppola and V. Kantsler
University of Warwick, Physics Department, CV47AL, UK

Scattering angle determination

Fig. 3 shows the most likely scattering angle θ_{out} for a given incoming angle θ_{in} at which CR approach the interface between the two regions. The relationship between the two angles was analysed by producing a bivariate histogram (Fig. S1), which allows us to determine the probability $p(\theta_{out}|\theta_{in})$ for any given scattering event. The probability heatmaps do not vary significantly for the different strains and interfaces used during our experimental investigation Therefore, we only show results for a wt CR swimming between regions of 0.0% MC and 0.30% MC ($6\eta_0$).
It is possible to notice from the bivariate histogram that CR approaching the interface from the low viscosity region will reorient and scatter off it, since the most probable θ_{out} is found to be negative for all θ_{in} smaller than $\sim \pi/6$ (positive angles indicate successful crossing from low to high viscosity). On the contrary, when CR approaches the interface from the high viscosity side, the most probable θ_{out} values all indicate successful crossing, regardless of the angle of approach θ_{in}.
The bivariate histogram allowed us to perform a statistical analysis of all the events for a given θ_{in}, from which the data for Fig. 4 was obtained.

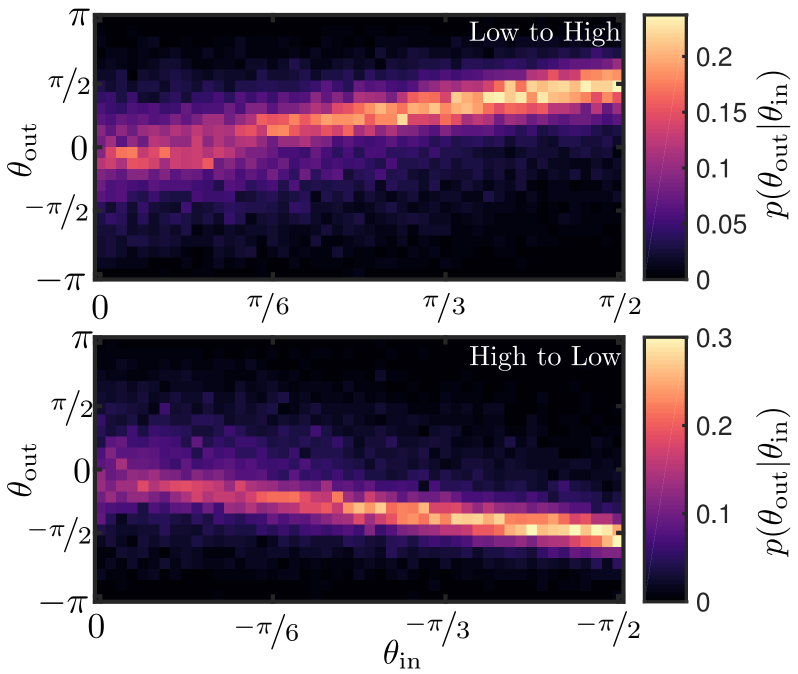

Figure S1. The bivariate histogram for scattering events for wt CR trying to cross from low to high viscosity (up) and high to low (down). In this case, $6\eta_0$ was used as the high viscosity medium.

Concentration Profile and Flux

Fig. 4 shows how the concentration profile of CR changes inside the chamber for all the different viscosity gradients we investigated. In particular, we highlighted CR can concentrate in the low viscosity region for some of the cases. In this section we further the discussion by showing more results on the subject. After tracking the algae it is possible to obtain probability heatmaps for their y-position as they evolve in time (Fig.S2). In the figure, it is possible to distinguish the difference in the effect between wt and sfl CR in the $6\eta_0$ case: wt concentrate significantly in the low viscosity region ($\nu = -0.5$), while the result is minor in the sfl case ($\nu = -0.1$).
We can further our analysis by calculating the flux of algae as a function of their y-position in the channel (Fig.S3). The flux through a certain y-position over all timesteps is given by the sum of all the Δp values, where Δp is the difference between the probability in the ith column and the previous one.
The results we find for the fluxes highlight further the findings from Fig.5: wt CR show a net positive flux towards the low viscosity region for the 2 and $6\eta_0$ cases, whereas the 19 and $62\eta_0$ result in a net zero flux. Similarly, only minor effects can be seen in the flux for the sfl experiments, thus showing that the algae concentration within the chamber remains uniform over the course of the experiment despite the significant swimming velocity ratio of the algae in the two regions.

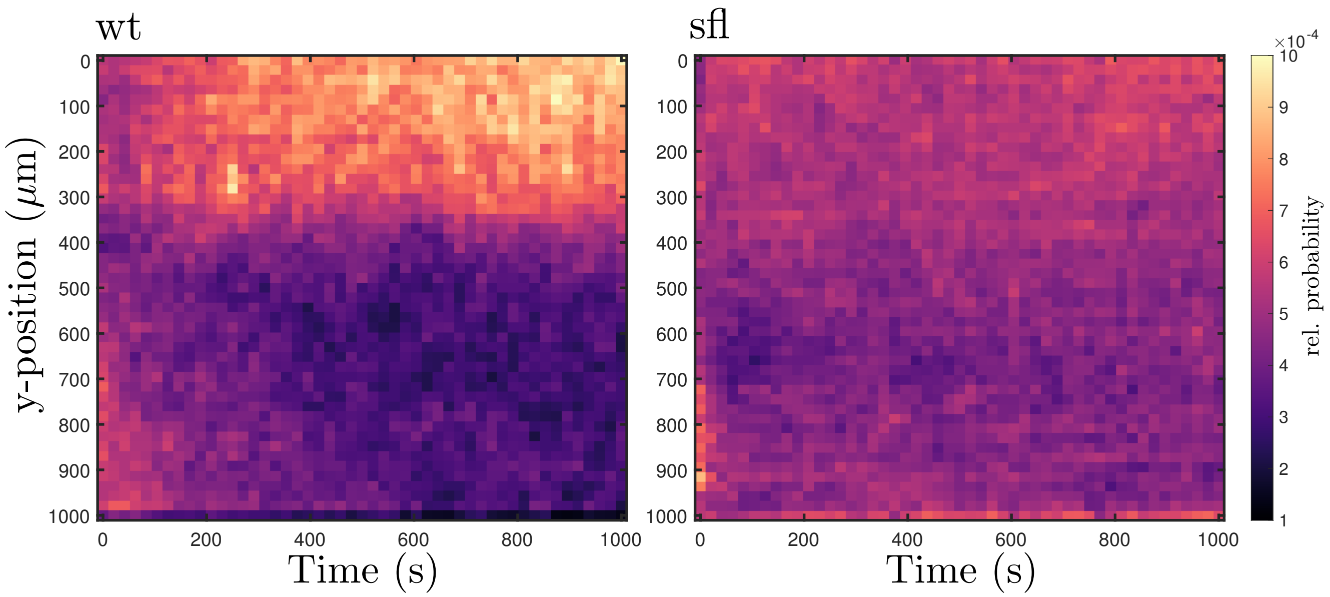

Figure S2. Probability heatmaps for wt (left) and sfl (right) CR in a device with a high viscosity region of $6\eta_0$. Both heatmaps were obtained by averaging multiple experiments. Colorbar is the same for both figures.

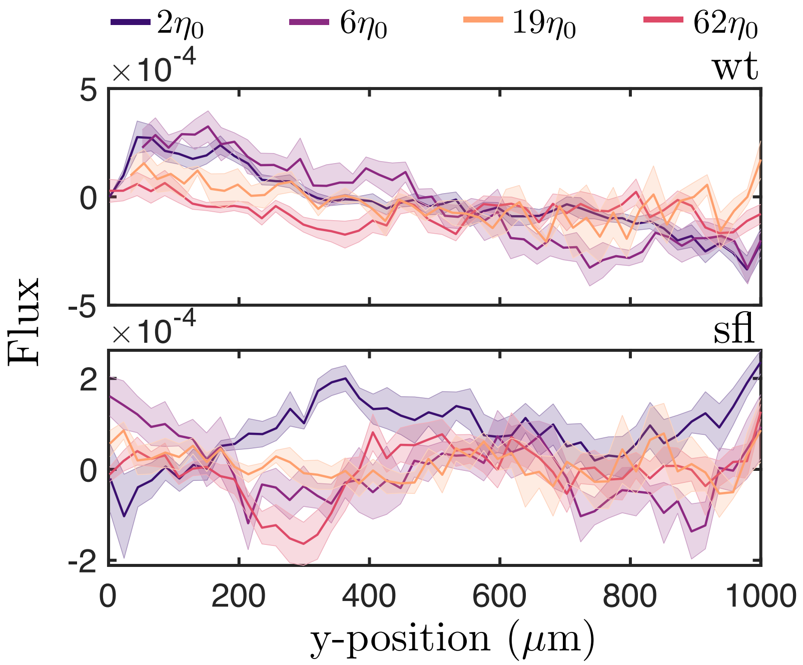

Figure S3. Fluxes as a function of the y-position in the chamber for wt (top) and sfl (bottom) for different viscosities. In all cases the low viscosity region corresponds to the left side of the figure, while the high viscosity region corresponds to the other half.
